# Understanding Emergency Department Healthcare Professionals' Perspectives of Caring for Individuals in Suicidal Crisis: A Qualitative Study

**DOI:** 10.3389/fpsyt.2022.918135

**Published:** 2022-06-13

**Authors:** Demee Rheinberger, Jessica Wang, Lauren McGillivray, Fiona Shand, Michelle Torok, Myfanwy Maple, Sarah Wayland

**Affiliations:** ^1^Black Dog Institute, University of New South Wales, Randwick, NSW, Australia; ^2^Faculty of Medicine and Health, University of New England, Armidale, NSW, Australia

**Keywords:** suicide, suicidal crisis, emergency department, qualitative method, healthcare professionals, nurses, lived experience

## Abstract

Help seekers regularly present to Emergency Departments (EDs) when in suicidal crisis for intervention to ensure their immediate safety, which may assist in reducing future attempts. The emergency health workforce have unique insights that can inform suicide prevention efforts during this critical junction in an individual's experience with suicide. This paper explores the treatment and care delivery experiences of 54 health professionals working in EDs within one of the LifeSpan suicide prevention trial sites in Australia. Data was collected via six focus groups and six interviews. Thematic analysis resulted in three themes: (1) physicality of the emergency department, (2) juggling it all–the bureaucracy, practicalities, and human approach to care, and (3) impact of care delivery on ED staff. Findings highlight the need for workplace training that incorporates responding to the uncertainty of suicidal crisis, to compliment the solution-focused medical model of care. Broader policy changes to the ED system are also considered to ensure better outcomes for health professionals and help-seekers alike.

## Introduction

Suicide accounts for 700,000 deaths globally each year (1). In Australia, it is recommended that individuals experiencing a suicide crisis (i.e., suicidal ideation or suicide attempt) present to a hospital emergency department [ED, (2)]. One in every 25 people who present to an ED for self-harm will die by suicide in the following 5 years, with non-fatal reattempt rates more than five times higher over the same period (3). Given that individuals in a suicide crisis typically attend the ED seeking immediate physical and psychological intervention to inhibit their suicidal actions (4) their care should address their immediate safety which may assist to reduce their risk of future attempts.

From a patient perspective, research has shown that a negative experience of care in ED results in individuals being less willing to engage with support services upon discharge (5, 6) and to return to the ED in a future suicide crisis (5). Negative experiences when presenting to the ED includes interactions with staff where communication is marred by stigmatizing attitudes and low empathy, staff emphasizing the delivery of medical care before psychological care, and excessively long wait times (7–9). Individuals presenting for suicidal crisis (herein referred to as help seekers) have observed fatigued and stressed staff, consistent with presentations of burnout (10). Due to the medical focus of ED treatment, presentations to the ED–including suicidal crises-prioritize the assessment of physical safety, which is likely a result of the vast majority of ED presentations being for physical concerns (11). Furthermore, help seekers report that staff appear to be ill-equipped with adequate knowledge of mental health and suicide (9).

From a healthcare perspective, some staff have previously reported negative views about those in suicidal crisis. Recent studies identified that ED nurses and doctors who report negative attitudes toward patients presenting in a suicide crisis or with intentional self-harm experience low empathy and some degree of antipathy toward mental health patients in general (12–14). Australian emergency nurses indicated that negative attitudes toward help seekers impacted the quality of care they delivered (13). ED staff also report not having time to build rapport necessary for a psychological assessment (15), or to appropriately assess the risk of future suicide (12, 15, 16). Moreover, research has suggested that ED staff are inadequately trained in mental health (17, 18) and suicide presentations, such as causes, crisis intervention, assessment, and appropriate referral options (12, 14, 19, 20).

Systemic issues within health services are also important considerations in the provision of ED care. ED health professionals report struggling to provide care due to not being able to access to essential resources, such as patient beds (13, 21, 22), and internal mental health professionals to lead mental health assessments and ensure specialized care of help seekers (20, 21, 23). EDs are high-pressure environments and interacting with people in suicidal crisis may further exacerbate existing levels of occupational stress amongst healthcare professionals in this setting (24, 25). Studies have found that engaging with help seekers can have a detrimental impact on the mental health of ED professionals, such as emotional exhaustion (26, 27), compassion fatigue (28, 29), and burnout (27, 30); all of which predict poorer quality of care for help seekers (31, 32). It is apparent that individuals in suicidal crisis are seeking help from health providers who have also reached their capacity. Understanding how and why ED staff are challenged by systemic factors to provide adequate care, will be crucial to addressing this bidirectional crisis, which is expected to be exacerbated by the COVID-19 pandemic. This information can help ensure that future health system reforms are not at the expense of staff capacity, capability, and wellbeing.

A comprehensive literature search did not yield any study that has explored the experience of caring for and treating individuals in a suicide crisis from the perspective of ED staff from multiple professional roles. Considering this gap, the aim of this study was to explore the experience of providing care to individuals in a suicide crisis from the perspectives of staff in a variety of roles within the ED to gain novel insights into key challenges preventing them from offering adequate care to individuals experiencing a suicide crisis.

## Materials and Methods

This study was approved by the Hunter New England Human Research Ethics Committee (HREC/17/HNE/144). Participants were provided with participant information sheets prior to their interview or focus group and written or verbal consent was obtained from all participants prior to the commencement of interviews and focus groups.

This study comprises one component of the qualitative data collected as part of a longitudinal cohort study, which has been reported elsewhere (33). In summary, the study explored the experience of using, and providing care through, the ED for suicide crisis via online survey with help seekers, as well as interviews with help seekers, carers, and ED healthcare professionals. The study is part of LifeSpan, a larger, multi-component suicide prevention trial (34), however, no interventions had been implemented with respect to EDs or ED healthcare professionals at the time of data collection.

### Participant Recruitment

ED staff (*N* = 54) were recruited via convenience sampling from two EDs (ED1 and ED2) within one of the LifeSpan trial sites in February and March 2021. Both EDs were within the same health district in a large regional metropolitan area and were both open to the public. ED1 was part of the local public hospital and had approximately 87,000 ED presentations in the year prior to the study taking place. ED2 was part of a large private hospital and saw approximately 55,500 ED presentations in the same period. The authors opted not to identify the hospitals further to protect confidentiality of participants. Focus groups were scheduled during established training periods, and an invitation was extended to all staff to participate in a focus group. Established training windows were used for the focus groups to reduce the impact of removing staff from the ED, and to ensure that staff from the same profession were grouped together to encourage open communication amongst established teams. All clinical ED staff were invited to participate in a one-on-one interview. Interview recruitment was managed internally via an ED specialist who worked at both hospitals and was involved in a local health working group focused on reducing suicide rates in the area. At the completion of the focus groups participants were also offered the option to continue the conversation via an interview at a later date.

Inclusion criteria were any staff who worked in one or both of the two EDs, in a patient facing role (e.g., nurses, registrars, etc.), there were no exclusion criteria. All participants had experience engaging with help seekers in the ED.

### Data Collection

Data were collected via six focus groups and six interviews (Table 1). Focus group and interview questions were semi-structured and designed to explore the participants' experiences of providing care to individuals presenting to the ED during a suicide crisis, with a focus on role and confidence providing care, standard of care and treatment, and barriers and facilitators to care.

**Table 1:** Focus group and interview demographics.

	**ED 1**	**ED 2**	**Total *N***	**Female *N* (%)**
**Focus group**				
ED Nurses	X		6	4 (67%)
ED Nurses		X	9	8 (89%)
Registrars	X		11	5 (45%)
SRMOs	X		5	3 (60%)
SRMOs		X	2	1 (50%)
Specialists	X		16	7 (43%)
**Interview**				
Mental Health Nurse	X			X
Mental Health Nurse	X			X
Psychiatric Registrar	X			X
Specialist	X	X		X
Specialist	X			X
Specialist^*^	X			X
	**Total** ***N*** **=** **54**

*SRMO, Senior Resident Medical Officer; ED, emergency department; ED1, larger public ED; ED2, smaller ED, connected to the private hospital*.

* *Indicates participation in both focus group and interview*.

Focus group participant numbers ranged from two to 16. Five focus groups were conducted face-to-face, with one conducted via video conference. Focus groups were approximately 60 min long, and staff were informed that participation was voluntary and that they were free to leave at any time. Focus groups were facilitated by the lead author and another member of the research team [ES] who had working knowledge of the local health system. Interviews were conducted over the phone by the lead author and ranged between 60 and 90 min in length. Interviews and focus groups were digitally recorded and de-identified before being sent to a secure third-party service for verbatim transcription.

### Analysis

A descriptive, inductive thematic analysis was undertaken to develop a broad understanding of the data (35). Staff experiences of providing care were coded from each transcript individually, using the Nvivo Software (version 12). The process of code development was repeated multiple times to ensure that all components of staff experience were understood. Codes were subsequently grouped into categories, and then broader themes. The process of code and theme development was undertaken by two authors (DR and JW) independently to ensure intercoder reliability. Any discrepancies were discussed between the two authors until consensus was reached. Codes were reviewed and final themes were arrived at through in-depth discussions between the two coders and a third researcher (SW, who was independent from the coding and data collection process), to reach consensus and to facilitate robust discussions on the inclusion of themes.

## Results

Thematic analysis resulted in three themes which explore the experience of providing care in the ED to help seekers presenting during a suicide crisis. Firstly, the physical environment of the ED provided some challenges for ED staff engaging with help seekers, particularly when this hindered the ability to provide compassionate care, or it increased help seeker distress. The second theme focused on the disconnect between the care that health professionals are expected to deliver, and the care they believe the help seekers need. The third theme explored the impact of care delivery on the ED staffs' mental health and wellbeing. A summary of these findings can be found in Table 2.

**Table 2:** Three identified themes and the subthemes within each.

**Theme**	**Theme**	**Theme**
Physicality of the Emergency Department	Juggling it all–the bureaucracy, practicalities, and human approach to care	Impact of care delivery on ED staff
- Chaotic and loud - The built environment limits privacy	- Rapport building is limited by insufficient time - Risk stratification and person-centred care - Limitations of the medical model - The ED needs to be a multidisciplinary team	- Feelings of futility - Staff burnout and the ongoing impact of the workplace

### Theme 1: Physicality of the Emergency Department

#### Chaotic and Loud

Staff described the ED as chaotic, busy, loud, and bright and recognized that these aspects all have a negative impact on someone in psychological distress, such as a suicide crisis.

“You've got people who are voluntarily seeking help for their suicidality, thoughts of self-harm, potentially waiting hours in a high stress environment. … Sitting here for half an hour, I'm sure you've heard multiple announcements, patients screaming, children screaming. It's not a pleasant place to be. I don't think this is really a good space for patients to get help.” – Registrar (FG, ED1).

Staff emphasized the need for a calm and safe space, dedicated to help seekers who present to the ED during suicidal crisis. However, often such places were limited or non-existent and staff felt forced to try to come up with solutions spontaneously.

“I think, by and large, most people are doing their best. Most people get that it's not ideal and try and work around it. You try and find somewhere quiet in the hospital. You use a family room, or you sneak them into one of the de-escalation rooms.” – Psychiatric Registrar (ED1).

#### Built Environment of the ED Limits Privacy

There is also little privacy for people being triaged in the ED, or once admitted to the ED. Staff reflected that it was often difficult to have sensitive conversations with help seekers about their suicide crisis as there was no private, “quiet spot” (Specialist – ED1) for them to talk. Staff commented that triage areas are open, where it is possible for other patients and staff to hear sensitive conversations. Similarly, once admitted to the ED there is little more than plastic curtains separating the help seeker from the patients in neighboring beds which again limits privacy during a difficult and distressing conversation.

“I don't think people should be getting reviewed and asked to talk about their deepest, darkest fears and insecurities or whatever, when everyone around them can hear it.” – Psychiatric Registrar (ED1).

The current physical environment of the ED is not appropriate for help seekers due to the intensity of noise and busyness, and the lack of privacy. This impinges upon the health professionals' ability to provide effective and timely care as they are pressed to take additional steps to rectify the environmental concerns before they can begin assessment or treatment of the help seeker.

### Theme 2: Juggling It All–The Bureaucracy, Practicalities, and Human Approach to Care

#### Rapport Building Is Limited by Insufficient Time

A consistent theme shared by the health professionals was their experience of the overwhelming impact of not having sufficient time to spend engaging with help seekers. ED staff proposed help seekers would likely benefit from this engagement. Staff recounted needing around 90 min to do a full assessment with someone experiencing a suicidal crisis, however many only had around 15 to 20 min to sit with a help seeker to understand their presentation and risk level.

“...You could spend an hour and a half with one patient whose [mental health] deteriorated …” – ED Nurse (FG, ED2).

“I do not do a 90-minute mental health assessment on these patients, which is what I know it can take a psychiatry senior registrar... it can take 90 minute, two hours sometimes to do that. That is not my job. My job is to try and ascertain the most pertinent elements of the history.” – Specialist (ED1 & ED2).

Almost all staff felt as though not having enough time to sit with help seekers led to less comprehensive assessments, and many feared that this would result in insufficient treatment plans, and potentially a re-presentation of the help seeker for a future suicide crisis.

“I think everyone would agree, if you had an infinite amount of time, you could spend it with them and you would get good outcomes every time. And you'd probably have faster plans and more efficient flow through the ED but the problem is that none of us can sit down for an hour and a half.” – Specialist (FG, ED1).

This is further exacerbated by a mandated key performance indicator (KPI) in which staff should see and treat a patient within 4 h from admission to discharge from the ED. ED staff felt strongly that this KPI was inadequate for providing care to patients who have complex presentations such as suicide crises, as many staff felt rushed to make a decision regarding the help seekers ongoing care or discharge.

“… The legislation mandates that they are seen within four hours. … generally, it's a cursory assessment being made as to whether they need to be kept under the Mental Health Act rather than any kind of compassionate care.”– Psychiatric Registrar (ED1).

However, ED staff worried that spending the necessary time with these help seekers limited the time they had available to other patients in the ED resulting in increased waiting times. Furthermore, staff were concerned that they would be unable to leave the help seeker in the middle of an in-depth assessment to attend to an unforeseen, life-threatening emergency.

#### Risk Stratification and Person-Centered Care

Staff discussed not feeling confident with the risk stratification process or in the efficacy of the suicide assessment tools available to them. This created a tension between the medical need for diagnosis, prediction, and certainty, and the uncertainty of complex and dynamic suicide risk.

“Every single patient is different, compared to any other medical condition. You cannot give a blue-print and say this is what you need to follow because unfortunately it doesn't work.”– Registrar (FG, ED1).

Additionally, staff recognized that no assessment tool could fully account for an individual's risk factor of future suicide death. This reduced their confidence to accurately risk stratify and deliver best possible care to help seekers.

“The trouble in psychiatry, particularly for suicidal risk, there isn't actually a validated tool that is good enough, that we can say ‘we've done these five things, therefore you are safe, therefore you can go.' Which disempowers us, because what that then leads to is this need for experience and familiarity, … and so many of us feel less comfortable that we're the right person, that it's not something that we're empowered very well to be able to do.” – Specialist (FG, ED1).

This was particularly pronounced when attempting to risk stratify help seekers who fell between the high-risk category (e.g., having made a suicide attempt, access to means, no social support) and the low-risk category (e.g., suicidal ideation without attempt, strong social supports, poor access to means or means easily restricted).

“It's extremely hard … [some] patients are clear cut, and you could have one look at them, put them on in order and you wouldn't look twice at it. And then there's a huge range of gray, which is extremely challenging to navigate.” – Senior Resident Medical Officer (SRMO, FG, ED1).

Difficulty with risk stratification was prevalent across all professional levels within the ED, even within the mental health professionals who spoke of trusting a “gut feeling” rather than always being able to empirically determine the level of risk or appropriate actions to take.

“There's No Really Good Suicide Tool in Terms of Risk Assessment, So That if I'm Worried About Someone, I Go With That. I Don't Ignore It.” – Psychiatric Registrar (ED1).

#### Limitations of the Medical Model

General ED staff reported a tendency to focus on the medical aspects, rather than any mental health or psycho-social aspects of suicide related presentations, which was often due to staff having more confidence engaging with physical aspects of medical care:

“99.9% of us and the medical staff are all more confident with the physiological [cases] because we're highly trained in that.” – ED Nurse (FG, ED2).

Staff spoke of “medicalising acute distress” (Specialist – FG, ED1) to attempt to reconcile the disconnect between providing physical treatment for an aliment and the intangibility of psychological distress. Help seekers who were deemed to be of low risk were often discharged soon after assessment and were often not transferred into the care of ED mental health team. To overcome difficulties proving care to individuals with greater risk, general ED staff would often refer the help seeker to the internal ED mental health team, which was a source of relief for many staff, who felt it was not their responsibility to make decisions about ongoing care:

“I'm grateful that we've got the mental health team because then the decision is out of my hand. Because I can't make that decision.” – SRMO (FG, ED1).

While in some cases (such as high suicide risk requiring admission to the psychiatric ward) this may be best practice for the help seeker, it limited opportunities for ED staff to develop confidence engaging with, and treating patients in a suicide crisis, and could result in longer waiting times for the help seeker.

#### The ED Needs to Be a Multidisciplinary Team

Despite the reliance on the internal ED mental health team to ensure appropriate treatment for help seekers, staff reported limited access to multi-disciplinary mental health staff in the ED. ED health professionals recognized that mental health trained staff could more effectively assess help seekers risk and make appropriate discharge/admission decisions. In addition, mental health professionals were seen as having more time to spend with help seekers due to different demands on their time, and metrics for their role.

“I think that what works well is having a designated staff member whose sole job is to assess mental health patients. That certainly takes a load off the medical staff who have less training and less resources at the disposal. … The best people looking after mental health patients are mental health clinicians”–SRMO (FG, ED1).

However, staff reported insufficient availability of mental health staff to respond to the number of mental health patients (including those in suicide crisis). Furthermore, mental health staff were limited to working within business hours on weekdays only, while the ED never closes.

“The limitation and the time that you need and services that are available, especially at night-time everyone knows that at night-time, what can you do for this patient? Nothing. … Whereas in the day you can see the patient quickly and then you can just call [the ED mental health team], they can come to see the patient, whereas if I have a patient at 11 o'clock, I know that the patient is going to stay here the whole night.” – SRMO (FG, ED2).

This resulted in some help seekers waiting hours to be assessed by a mental health professional in the ED, which puts a strain on ED staff who have to ensure that care of these individuals is continued for much longer than is typical for ED patients;

“… probably the greatest difficulty I have in dealing with suicidal patients is probably on night shifts and being able to just deliver compassion and care to them.” – Registrar (FG, ED1).

ED staff viewed the healthcare metrics of the ED as inconducive to providing care for help seekers, as well as impacting their ability to provide high quality care. Low confidence led many participants to focus on physiological over psychological presentations, and to refer the treatment, assessment, and discharge of complex help seekers to mental health ED staff.

### Theme Three: Impact of Care Delivery on ED Staff

#### Feelings of Futility

ED staff raised concerns about individuals in suicidal crisis receiving inadequate care, despite staff doing the best they can within the constraints of the ED. This was reaffirmed when help seekers presented to the ED in a suicide crisis on multiple occasions; staff reported feeling that any previous time or intervention they had provided to that individual was pointless, and that their efforts had been in vain.

“At the end of the day, you feel like you … there is a lot of knowledge there, you try to learn a lot, but at the end of the day you can't do anything for these people. It's a point of frustration. So, you spend two, three hours and then at the end of the day you can support them, but there's no treatment.” – SRMO (FG, ED2).

“I feel like, in the ED at least, we sort of put band aids on … problems that can't be solved in the emergency department.” – SRMO (FG, ED1).

ED staff reflected that the issues which lead to a suicide crisis are driven by social issues which are beyond the scope of what can be altered within an ED visit, further exacerbating the feelings of futility and powerlessness;

“But the tsunami of not coping is really difficult here, because we just feel completely powerless... ‘How can we help you?' My gut feeling is how do we reform the social situation that you were brought up in to lead you to this? We can't, so therefore we will see you again next week. … For some of them it's a very revolving door feeling. I actually don't think the solution is in health care, to be quite honest.” – Specialist (FG, ED1).

“… is it something financial, is it an abusive partner? We just don't have that resource as clinicians to then fix what's actually led them to feel that way. … I can offer that comfort, but I can't actually fix the attributes, unfortunately.” – Registrar (FG, ED1).

#### Staff Burnout and the Ongoing Impact of the Workplace

Ultimately, ED staff expressed a very strong desire to assist help seekers, however, were conflicted by feelings of futility and powerlessness which were consistently compounded with the re-presentation of help seekers. For many participants this led to frustration and reduced empathy toward help seekers;

“That's a big problem, too. Because we're so stressed out, our mental health is not great either a lot of the time. When your mental health is not great, you've got nothing left to actually help someone with …” – ED Nurse (FG, ED1).

“Honestly, I just think that's the burn. I don't think people are bad people, I think people just get burnt out and it's just frustration. … It is a big problem, and the thing is just that once people start to burn out, their own self-defenses have to kick, so people get cynical, and they get desensitized.” – Psychiatric Registrar (ED1).

ED staff spoke of having struggled with the professional concerns that arise due to experience of working with help seekers, such as burn out, emotional exhaustion, and trauma (vicarious and witnessed).

“Those suicides that I spoke about all occurred within two weeks of each other so there were three in two weeks. … I found that I was starting to ruminate and that sort of thing about one of them. … But I guess the thing is that a violent death in a young person is always really distressing, irrespective of the cause and we're dealing with those things pretty often.” – Specialist (ED1).

“There's some pretty harrowing stories that come through. I think you develop a little bit of armor that would come naturally … I'm sure it contributes quite greatly to part of the burnout that emergency physicians are very well known for.” – Specialist (ED1).

Despite these challenges, ED staff were only provided access to Employee Assistance Program (EAP) counselors, who provide support to staff across the hospital irrespective of the nature of their work (i.e., accountants, administrators, doctors, etc.). These EAP counselors are not specialized to assist staff with the traumatic challenges of working within an ED, which resulted in poor uptake by ED staff who felt that the formal sources provided by the hospital were ineffective. One health professional found the EAP counselor so unhelpful that they instead opted to engage with a private psychologist to provide assistance for the difficult aspects of working in the ED and with help seekers.

“There's the EAP, which is provided by both the hospital, and then our college also provides it. So, that's that. I have to say that occasionally I've used EAP in the past and I haven't found it particularly helpful. I have my own psychologist who I have been engaged with for many years, due to various reasons, and I firmly believe actually that emergency doctors should... Because we see some crazy stuff.–Specialist (ED1 & ED2).

Staff reported that peer support and comradery, which they used regularly to deal with the difficult aspects of their work, was useful in terms of engaging with informal support strategies. Staff recognized that this informal support helped mitigate the emotional exhaustion and burnout and advocated for more internal encouragement of seeking informal support.

“As we handover, people tend to stay back a little bit just to be able to kind of talk. So we kind of look after each other in that respect … And that needs to be done because I think, in terms of our own mental health, like what I have noticed is there's not a lot of staff retention, there's a lot of burnout, and there's a lot of people moving from job to job. And I think it's a no brainer that that's very much a symptom of a service that isn't supporting the people.”–Mental health nurse (ED1).

ED staff experienced several mental health impacts from attempting to provide care to help seekers whilst also operating within the constraints of the ED system. Most notably, ED staff reported feeling futile about addressing help seeker needs, which they believed were rooted in broader social issues and therefore beyond the scope of their role as health professionals in the ED. The pervasive feelings of futility alongside a strong willingness to help resulted in many ED staff feeling burned out. Furthermore, participants reported finding formal avenues of support (such as EAP) ineffective and were unlikely to engage in such services, instead preferring more informal avenues of support.

## Discussion

The experiences of ED staff who care for help-seekers presenting in suicide crisis can offer strategies to guide emergency health system reforms that can have mutual benefits for staff experiences and patient outcomes. This study sought to explore and understand the experiences of ED staff when they are providing care to individuals in a suicide crisis. As such, the qualitative analysis of focus groups and interviews revealed three core themes that explored aspects of working in the ED which impacted care for suicide patients: (1) aspects of the built environment (described as loud and chaotic) that may not be conducive to helping people in crisis; (2) the disconnect between the bureaucracy, practicalities of working within the ED, and the human approach to care (for instance not having enough time with help seekers, low confidence with risk stratification, and poor access to mental health staff); (3) consequences of care delivery on ED staff (such as feelings of futility, burnout, and lack of effective formal support).

Recent literature has shown that an ED visit is often one part an individual's suicide-related journey across their lifetime (36) and is a common gateway for individuals to access the mental health system within a primary health or community setting. Certain healthcare metrics in the ED, such as the 4-h KPI to discharge patients, were felt by participants to be counterproductive to the care that help seekers required and restricted the degree to which healthcare professionals could establish rapport with these individuals. This view is supported by research that has demonstrated the 4-h KPI from admission to discharge does not improve patient outcomes (37, 38). Rather, a maximum 12-h length of stay is recommended to be built into KPIs to allow appropriate time to engage with mental health patients and limit the long waiting times experienced by help seekers (39). These healthcare metrics, such as the tight KPI, have a negative immediate impact on both the health professionals' ability to provide care and help seekers' experiences of their ED visit (9, 40), as well as a long-term impact by discouraging help seekers from accessing other avenues of mental health support in the future (5). ED staff identified that the physical ED space, lack of access to mental health professional staff, and inappropriate internal policies and procedures all contributed negatively to the care experience of help seekers.

ED staff in this study recognized that many of the issues that were leading help seekers into crisis were psychosocial, outside the control of the ED, and incompatible with the physiologically focused approach to care which ED staff are trained to deliver. This limited control was further exacerbated by ED staffs' limited confidence in their ability to stratify risk, which is not surprising given the complexity of suicide presentations. A 2021 systematic review of 21 studies showed that no one suicide risk assessment tool, including clinical assessment, could accurately predict the risk of future suicide behavior (41). In contrast, there is some evidence to show that greater exposure to help seekers may improve accuracy of assessed suicide risk (16), yet ED staff in this study often chose to relinquish this responsibility and defer to other ED mental health staff whom they believed were best placed to make assessment, treatment, and discharge decisions. Future research should explore and evaluate possible education and training options to better equip ED health professionals to engage with help seekers. Additionally, future reform efforts should consider providing training which integrates a compassion-focused approach to help seekers' distress and encourages staff to accept the uncertainty which comes with mental health and suicide crisis presentations to the ED.

Staff burnout appears to be linked to poor patient outcomes for help seekers (e.g., re-presentations, repeat attempts, or suicide death) (27, 30) and while the high incidence of burn out in ED staff is well documented in the literature (20, 26, 27, 42), mechanisms that contribute to burn out in relation to help seekers have not been explored. A recent meta-synthesis found that after the death of a help seeker, ED staff experience negative emotions such as shame and self-blame (43). Health professionals in this study spoke of being worried about the outcome of the help seekers in their care, choosing to relinquish responsibility of caring for them to alleviate feelings of inadequacy and self-blame should they attempt suicide in future. Staff experiences of emotional exhaustion and feelings of futility are influenced by the high rates of re-presentation of help seekers to the ED in suicidal crises (44). This re-presentation of suicidal crises has been linked with job dissatisfaction and burn out of ED staff (30) and in this study, was attributed to an inability to provide effective care in the ED for people experiencing suicide crisis. This finding is consistent with previous studies showing that, burned out staff are less likely to provide positive and compassionate care to help seekers (31, 32), and are more likely to transfer or resign from positions in the ED (26, 27, 45), which is problematic given that ED nurses with longer employment histories (and more experience) are more likely to exhibit positive attitudes toward help seekers (46, 47), and assess suicide risk more accurately (16). To prevent burn out and premature job loss, formal sources of mental health support should be made available to ED staff (e.g., specialized mental health services which can address the unique stresses and trauma experienced in the ED), yet the provisions available to ED staff were reported to be inadequate.

This study demonstrates the substantial systemic barriers to providing quality care for help seekers and that this contributes to a sense of futility and burnout among ED professionals. The concerns raised by ED staff in this study are consistent with those reported by help seekers themselves such as difficult physical environment, and poor access to mental health staff (9), which further supports the validity of these issues and the urgent need to reform EDs to support help seekers in suicidal crisis. Furthermore, addressing some of these systemic problems is likely to have a positive impact on ED staff mental health (i.e., burn out) and employment retention. Broader recognition of the vast systemic changes that are needed to improve the ED has prompted researchers, governments, and service providers to question whether the ED is an appropriate service for mental health presentations and suicidal crises. Alternatives to the “failed system” have been explored in Australia [e.g., (48, 49)] and the United Kingdom [e.g., (50)] but robust evaluations are needed, as the ED remains an important part of the service landscape for the foreseeable future, given it is the only 24/7 option and the need to treat physical injuries and self-poisoning. In addition to exploring alternatives to the ED, future research should also evaluate the appropriateness of dedicated mental health spaces within the ED, such as those similar to the specialized Psychiatric Emergency Service in the United States (51) or the Psychiatric Emergency Care Centres in Australia (52). As concerted efforts are made toward improving quality of care in EDs and creating viable alternatives to the ED, it is essential that the experiences of professional staff are considered in developing reforms to ensure that the disconnect between the care that ED staff are expected to deliver and the care they know help seekers need is addressed.

There are several limitations that should be considered. Firstly, the data do not include the experiences of any individual working within the mental health clinician role. This was largely due to the scarcity of mental health clinicians working in the ED. A psychiatric registrar and mental health nurses were interviewed, and their experiences may provide some insight into the experiences of mental professionals; however, input from mental health clinicians would be valuable. Secondly, the proportions of each role included in this study may not be reflective of the ratios found in EDs (for instance the lower percentage of registrars, and high number of specialists involved in the study). Further investigation is warranted to determine if these findings are representative of ED staff experience. Thirdly, researchers were unable to confirm the number of participants attending each focus group ahead of time, as ED staff were required to attend emergencies in the ED at any moment, resulting in some focus groups being smaller or larger than the six to 12 participants which has been recommended (53). This may have impacted the depth of the topics discussed by reducing time for deep exploration of ideas or limiting sense of anonymity typically achieved with larger focus groups (53). Finally, the proportion of participants from ED1 was greater than those from ED2. This may have been due to the differing capacity of the hospitals resulting in greater overall numbers of ED staff at ED1. While we may not have been able to capture a well-rounded experience of providing care to help seekers in ED2, the consistency across all focus groups and interviews in this study as well as with recent international research (20, 47) suggests that the information we collected is representative of the wider service delivery.

This study identified several built environment and procedural challenges that adversely impact the care that ED staff are able to provide to individuals in suicidal crisis, and the impact this has on the mental wellbeing of ED staff, contributing novel insights to prior research which focuses on attitudinal barriers and the patient experience. Although our findings suggest that health professionals are highly motivated to address help-seeker needs, current ED spaces, procedures, and resourcing do not facilitate this, leading many to experience feelings of futility about their role in providing care to help-seekers. Improving both the physical and procedural features of their work environment could provide mental health benefits for ED staff as well as the help seekers under their care. Future workplace training should encourage health professionals to accept the uncertainty of suicidal crisis, in conjunction with focusing on a “solution-focused” medical model of care. Broader policy changes (such as increased resourcing, and re-evaluating KPIs) to the ED system also need to be considered to ensure better outcomes for health professionals and help-seekers alike.

## Data Availability Statement

The raw data supporting the conclusions of this article will be made available by the authors, upon request, without undue reservation.

## Ethics Statement

The studies involving human participants were reviewed and approved by Hunter New England Human Research Ethics Committee. The participants provided their written and/or verbal informed consent to participate in this study.

## Author Contributions

DR, FS, MT, and MM: conceptualization. DR and FS: methodology, resources, and project administration. DR, JW, and SW: validation and data curation. DR and JW: formal analysis. DR: investigation. DR, JW, LM, and SW: writing–original draft preparation. DR, JW, LM, FS, MT, MM, and SW: writing–review and editing. LM and FS: supervision. All authors have read and agreed to the published version of the manuscript.

## Funding

The LifeSpan study and the cohort study were supported by funding from the Paul Ramsey Foundation and ACT Health.

## Conflict of Interest

The authors declare that the research was conducted in the absence of any commercial or financial relationships that could be construed as a potential conflict of interest.

## Publisher's Note

All claims expressed in this article are solely those of the authors and do not necessarily represent those of their affiliated organizations, or those of the publisher, the editors and the reviewers. Any product that may be evaluated in this article, or claim that may be made by its manufacturer, is not guaranteed or endorsed by the publisher.
